# Does Regular Exercise without Weight Loss Reduce Insulin Resistance in Children and Adolescents?

**DOI:** 10.1155/2013/402592

**Published:** 2013-12-12

**Authors:** YoonMyung Kim, HaNui Park

**Affiliations:** ^1^University College, Center for Heath and Wellness, Yonsei University International Campus, Incheon 406-840, Republic of Korea; ^2^Department of Physical Education, Yonsei University, Seoul, Republic of Korea

## Abstract

Despite considerable efforts to tackle childhood obesity, it is recognized as one of the biggest health problems globally. Childhood obesity is a leading cause of many comorbid conditions such as metabolic syndrome and insulin resistance as well as type 2 diabetes. A strong body of evidence suggests that regular exercise without calorie restriction or weight loss is associated with reduced insulin resistance as well as improved insulin sensitivity in overweight and obese adults. However, despite the well-known benefits associated with regular exercise alone, the independent role of exercise training without calorie restriction on insulin resistance is still uncertain in youth. Some studies observed that both the aerobic and resistance type of exercise training without calorie restriction resulted in meaningful changes in insulin sensitivity, suggesting that exercise alone is an effective therapeutic strategy for reducing insulin resistance in overweight and obese youth. However, only few studies are available on the optimal dose of exercise training without calorie restriction or preferred exercise modality for reducing insulin resistance, which warrants further investigations in the pediatric population.

## 1. Introduction

The prevalence of childhood obesity has been increasing throughout the world during the past few decades [1]. According to the recent report by the National Health and Nutrition Examination Survey (NHANES), more than one-third of children and adolescents aged 6–19 years are considered at risk for overweight (BMI ≥ 85th percentile) or overweight (BMI ≥ 95th percentile) in the USA [2]. Childhood obesity is recognized as a major public health concern since the simultaneous increase in obesity is paralleled by an increased prevalence of impaired glucose tolerance [3], metabolic syndrome (MetS) [4], and type 2 diabetes [5, 6] in youth. The overall prevalence of MetS, determined by the National Cholesterol Education Program-Adult Treatment Panel III (NCEP-ATP III) definition, was approximately 4.2% in the US children and adolescents [4], which was present in 28.7% in overweight youth (BMI ≥ 95th percentile) but only 0.1% in those with BMI ≤ 85th percentile [7]. Furthermore, it is well established that obese youth are more insulin resistant than their leaner counterparts [8, 9] and more likely to develop diabetes than those with normal weight [10].

Independent of ethnicity, age, and sex, obesity represents the most important risk factor for insulin resistance as well as increased circulating levels of insulin, leading to decreased insulin sensitivity and impaired *β*-cell function, thereby developing type 2 diabetes in youth [11]. In addition, earlier onset of insulin resistance may be afflicted with the increase in cardiovascular morbidity and mortality risks in adult life [12]. Therefore, it is important to develop effective strategies to prevent or treat obesity as well as insulin resistance in the pediatric population.

In adults, a number of well-controlled studies have reported that insulin resistance is improved with regular exercise training even without calorie restriction or weight loss [13–16]. Although the mechanisms for the beneficial effects of regular exercise training alone have not been fully understood, improved insulin sensitivity and glucose uptake induced by muscle contraction after exercise training seem to play an important role in alleviating insulin resistance [17, 18]. Although some intervention studies in youth [19–22] have also reported significant improvements in insulin resistance after exercise training alone (without calorie restriction or weight loss), the independent role of exercise training alone as a therapeutic strategy to reduce insulin resistance has not been firmly established yet. Furthermore, very little attention has been directed toward the impact of resistance exercise training alone on insulin resistance in children and adolescents.

Thus, in this review, we present the available evidence regarding the role of exercise training without calorie restriction for reducing insulin resistance in children and adolescents. In addition, we distinguish the exercise modality for enhancing insulin sensitivity, which should be recommended for the treatment of insulin resistance in this age group.

## 2. Physical Activity, Fitness, and Insulin Resistance

In adults, it is well established that physical activity and cardiorespiratory fitness are significantly associated with the constellation of diabetes-associated risk factors [23] as well as cardiovascular disease risks [24]. Thus, increased physical activity has a beneficial effect on reduction in the diabetes-associated risks by enhancing insulin sensitivity [25, 26]. Consistent with the observations in adults, cross-sectional studies in youth [27–29] have also shown that increased physical activity is associated with higher insulin sensitivity. Schmitz et al. [27] examined the association of questionnaire-determined physical activity and insulin sensitivity, assessed by the hyperinsulinemic euglycemic clamp technique known as the criterion measure of *in vivo* insulin sensitivity, in nondiabetic children aged 10–16 years (*n* = 357). They observed significant relationships between physical activity and lower fasting insulin (*r* = −0.12) and insulin sensitivity (*r* = 0.18) even after accounting for BMI, percent body fat, and/or waist circumference. In a recent report by Thomas et al. [28], accelerometer-determined physical activity, less prone to error than questionnaire, was also positively associated with intravenous glucose tolerance independent of fat free mass and race in black and white adolescents aged 12–18 year old.

With regard to the relationship between cardiorespiratory fitness and insulin sensitivity, previous cross-sectional studies in youth have reported conflicting results. Some studies [30–32] have suggested that cardiorespiratory fitness is not an independent predictor of insulin sensitivity when total or abdominal adiposity was taken into account, suggesting that fatness is of greater importance as a determinant of insulin sensitivity than cardiorespiratory fitness in youth. Conversely, other studies [29, 33–36] have demonstrated that cardiorespiratory fitness is inversely associated with insulin sensitivity even after accounting for body adiposity, suggesting that the negative effects of body fatness on insulin sensitivity may be mediated by the degree of cardiorespiratory fitness in youth. Although these conflicting results may be partially explained by the different subject characteristics or assessment techniques for insulin sensitivity and cardiorespiratory fitness, it is still inconclusive which component is a better contributor to insulin sensitivity in children and adolescents, which warrants further investigations.

It is well known that a single bout of aerobic exercise with sufficient duration and intensity can increase insulin-stimulated glucose uptake in contracting skeletal muscles and insulin sensitivity via increased translocation of glucose transporter (e.g., GLUT4) to the cell surface [17, 18]. However, studies have also reported that the positive effects of acute exercise on insulin sensitivity only persisted for about 48–72 hours [17]. In addition, detraining or inactivity can reduce the exercise-induced enhancement of insulin sensitivity in skeletal muscles in adults [37]. In youth, improved insulin sensitivity was also observed after a single bout of moderate-intensity (75% peak heart rate) exercise, which lasts at least 17 hours [38]. These observations suggest that exercise should be performed on a regular basis for the long-term improvement in insulin sensitivity.

A single bout of resistance exercise is also known to enhance insulin sensitivity at least 24 hours after the last exercise session in healthy individuals [39]. However, only limited evidence is available on this issue in the pediatrics, and the mechanisms by which the acute resistance exercise improves glucose regulation, as observed after the acute aerobic exercise, are not clearly understood, thereby requiring further investigations.

Combined together, the above observations clearly suggest that people should add regular exercise in their daily routine to increase or at least to maintain insulin sensitivity, which may further reduce the increased incidence of insulin resistance or associated risk factors in children and adolescents.

## 3. Aerobic Exercise and Insulin Resistance

Although many interventional studies have focused on the beneficial impact of exercise and calorie restriction-induced weight loss on insulin resistance and body composition, growing evidence also suggests that exercise training without substantial weight loss can induce meaningful improvements in insulin resistance in adults [13–16]. Duncan et al. [16] previously showed that a 6-month period of aerobic exercise training (3–7 days/week, 30 min/session, ~75% of heart rate reserve) without weight loss significantly increased insulin sensitivity, accessed by the frequently sampled intravenous glucose tolerance test (FSIVGTT) in sedentary adults. The change in insulin sensitivity was also inversely correlated with BMI (*r* = −0.48) in this study [16]. Although it is still questionable if the enhanced insulin sensitivity after aerobic exercise training depends on concomitant changes in total or abdominal adiposity or on the residual effects of the last exercise session, it is plausible that regular exercise alone significantly alters total or abdominal adiposity, particularly visceral adipose tissue, thereby influencing positive changes in insulin sensitivity.

Despite extensive observations and experiments in adults demonstrating the beneficial effects of aerobic exercise alone on insulin resistance, only limited evidence is available on this issue in children and adolescents (Table 1). Nassis et al. [40] examined the effect of a 12-week aerobic exercise training (3 days/week, 40 min/session, HR ≥ 150 bpm) without weight loss on insulin sensitivity in overweight and obese girls aged 9–15 years. After the exercise training, the authors observed a significant reduction in the insulin area under the curve (insulin AUC) following an oral glucose tolerance test (OGTT) by 23.3% without changes in body weight or total adiposity. Similarly, a recent study by Van Der Heijden et al. [41] also demonstrated that a 12-week period of moderate aerobic exercise training (2 days/week, 30 min/session, ≥70% of VO_2_ peak) resulted in decreased insulin resistance in postpubertal obese adolescents. Indeed, the decreased fasting insulin was significantly correlated (*r* ^2^ = 0.40) with the decreased visceral adiposity in this study.

To the best of our knowledge, there are 6 randomized controlled trials wherein the effects of aerobic exercise on insulin sensitivity were examined in children and adolescent (Table 2). Meyer et al. [42] reported that a 6-month period of aerobic exercise training (3 times/week, 60~30 min/session) without calorie restriction resulted in a significant decrease in fasting insulin (21%) in obese adolescents aged 11–16 years. Recently, Davis et al. [21] compared dose-response effects of the short-term (13 weeks) aerobic exercise training (5 times/week, low dose: 20 min/session versus high dose: 40 min/session) on insulin resistance in overweight and obese youth aged 7–11 years with the exercise intensity held constant at a moderate-to-vigorous level (HR ≥ 150 bpm). After the exercise training, the decrease in insulin AUC was significantly greater in both exercise groups than the controls although the magnitude of the decrease in insulin action and adiposity was somewhat greater in the high-dose exercise training group versus low-dose exercise training group. These observations suggest that low- and high-dose of moderate-to-vigorous physical activity in most days are similarly effective in improving insulin resistance in overweight and obese youth.

Savoye et al. [19] examined the influence of 1-year behavioral modification program combined with nutrition education and aerobic exercise (1–6 month: 2 times/week, 50 min/session, 7–12 month: 2 times/month, 100 min/session, 65–80% of maximal HR) on body composition and insulin resistance in overweight youth aged 8–16 years. The authors reported significant decreases in fasting insulin levels (−28.3%) and homeostasis model assessment of insulin resistance (HOMA-IR, −29.8%) in the exercise training group as compared to the controls after the intervention. Furthermore, these improvements were sustained at 12 months of the intervention in the exercise training group.

Taken together, these observations provide some evidence that engaging in regular aerobic types of exercise in the absence of weight loss or calorie restriction is beneficial to improve insulin resistance in overweight and obese youth.

## 4. Resistance (or Combined) Exercise and Insulin Resistance

Although majority of studies have implemented aerobic exercise training as an efficient exercise modality for reducing insulin resistance, considerable evidence also suggests a beneficial effect of carefully supervised resistance (or combined) exercise training without calorie restriction on insulin resistance in adults [43–45]. Davidson et al. [45] reported that resistance or combined exercise training without weight loss or calorie restriction is associated with a significant improvement in insulin sensitivity in overweight or obese adults, which may be also attributed to increased fat free mass (muscle mass) or decreased total or abdominal fat mass after resistance training. However, relatively little evidence is available on the impact of resistance exercise training alone on insulin resistance in overweight youth. Indeed, it is still unclear if resistance type of exercise training is more effective than aerobic type of exercise training to improve insulin resistance particularly in overweight or obese children and adolescents.

Previously, Treuth et al. [46] have demonstrated that although modest changes were observed, a 5-month resistance training (3 times/week, 20 min/session, >50% of 1-repetition maximum) did not significantly alter fasting insulin (−4.1%) or fasting glucose levels (−4.0%) in obese girls aged 7–10 years (Table 3). Conversely, using the clamp technique, Bell et al. [47] reported that an 8-week period of combined exercise training (3 times/week, 60 min/session) without weight loss was associated with a significant improvement in insulin sensitivity (22.2%), determined within 48 hours after the last training session in obese youth. The improvement in insulin sensitivity was correlated with the improved cardiorespiratory fitness levels but not with body composition changes, suggesting that fitness level is of importance to reduce or prevent insulin resistance in obese children and adolescents. However, given the combined exercise training format applied in this study [47], it is limited to distinguish if the improvements may be solely due to resistance exercise training in this population.

To the best of our knowledge, there are 6 randomized controlled studies wherein the effects of resistance (or combined) exercise training without calorie restriction on insulin sensitivity were examined in children and adolescent (Table 4). Shaibi et al. [22] examined whether resistance training alone is effective for improvements in insulin sensitivity in overweight Latino male adolescents (*n* = 22). The subjects were randomly assigned to either resistance training group (2 times/week, 60 min/session) or control group. After the intervention, insulin sensitivity, determined by FSIVGTT, significantly increased (45.1%) in the exercise group, as compared to the controls. In this study, the increased insulin sensitivity was induced independent of cardiorespiratory fitness or body composition changes. Recently, Lee et al. [20] conducted a randomized controlled trial examining the effects of aerobic exercise versus resistance exercise without calorie restriction on insulin sensitivity in black and white obese male adolescents aged 12–18 years. They demonstrated a significant improvement in insulin sensitivity (27.6%), assessed by the euglycemic clamp technique, after a 3-month resistance exercise training (3 times/week, 60 min/session) without calorie restriction but not after the aerobic exercise training. Unlike the observations by Shaibi et al. [22], the improvements in insulin sensitivity was associated with exercise-induced increases in skeletal muscle mass as well as cardiorespiratory fitness levels in the resistance exercise training group in this study [20]. Furthermore, when both exercise groups were combined, the improvements in insulin sensitivity was significantly correlated with decreased total adiposity (*r* = −0.43) and visceral adipose tissue (*r* = −0.42), suggesting that improvements in body composition may play an important role to enhance insulin sensitivity in obese adolescents. In addition, the authors also noted that as compared with aerobic exercise, resistance exercise may be a better exercise modality to enhance body composition and insulin sensitivity particularly in obese male adolescents.

Taken together, despite the lack of conclusive evidence in the pediatrics, the observations demonstrating that resistance exercise training is associated with improvement in insulin resistance are clearly encouraging children and adolescents to participate in resistance type of exercise on daily basis. However, future studies are also required to explore the long-term effects of resistance exercise training on insulin resistance as well as determining the optimal exercise modality, as the effective therapeutic strategy to reduce insulin resistance in this population.

## 5. Summary 

The dramatic increase in childhood obesity in recent years is paralleled by decreased physical activity as well as cardiorespiratory fitness levels, which is also attributed to increase insulin resistance and diabetes-associated risk factors in children and adolescents. As observed in adults, an increasing body of evidence suggests that acute physical activity and chronic exercise have a beneficial effect on reductions in insulin resistance through multiple adaptations such as improved insulin sensitivity and glucose uptake of skeletal muscles and body composition changes in overweight children and adolescents. Studies also demonstrated that both aerobic and resistance type of exercise alone (without weight loss or calorie restriction) resulted in significant improvement in insulin sensitivity, suggesting that exercise alone is an effective therapeutic strategy for reducing insulin resistance in overweight and obese children and adolescents. However, future studies are required to explore the optimal dose of exercise and efficacious modality to elicit meaningful reductions in insulin resistance in youth.

## Figures and Tables

**Table 1:** Aerobic exercise and insulin resistance (nonrandomized controlled trials).

References	Gender	Age	Treatment	BW (kg)	BMI (kg/m^2^)	IS	Study Duration	Protocol	ΔBW (kg)	ΔBMI (kg/m^2^)	ΔIS (%)	IS Measure
Nassis et al. [40]	19 girls	9–15	Exercise	67.9	26.8	4.34	12 wk	3 days/wk, 40 min/day running, steps, stair climbing, and so forth HR > 150 bpm	0.4	−0.1	1.1	HOMA-IR

Caranti et al. [48]	37 boys 46 girls	15–19	Exercise	102.8 92.5	36.2 35.7	4.8 3.6	1 yr	Exercise combined with nutrition, psychological, clinical therapy 3 days/wk, 60 min/day (walking, cycling, etc.)	−10.5* −4.9*	−4.1* −2.1*	−33.3* −13.6	HOMA-IR

Monzavi et al. [49]	60 boys 49 girls	8–16	Exercise	78.2	33.7	5.52	12 wk	Lifestyle intervention program 90 min/session (weekly) 45 min of exercise (dodge ball, jump rope, etc.) + 45 min of nutrition education	0.1	−0.5*	−32.6	HOMA-IR

Kelishadi et al. [50]	19 boys 16 girls	12–18	Exercise	57.1	25.3	5.4	6 wk	3 days/wk, 60 min/day (30 min fitness + 30 min of games)	−2.4*	−1.2*	−22.2*	HOMA-IR

Van der Heijden et al. [41]	17 boys 12 girls	15.1	Obese exercise Lean exercise	91.7 57.2	33.7 20.6	4.9 1.7	12 wk	2 days/wk, 30 min/day ≥70% of VO_2_ peak treadmill, elliptical, cycle	−0.5^†^ 0.8*	−0.4^†^ 0.1	−16.3^∗†^ 11.8	HOMA-IR

BMI: body mass index; BW: body weight; HOMA-IR: homeostasis model assessment of insulin resistance; IS: insulin sensitivity; Δ: Change score.

*Significantly different from baseline within each group (*P* < 0.05).

^†^Significantly different from controls or other groups (*P* < 0.05).

**Table 2:** Aerobic exercise and insulin resistance (randomized controlled trials).

References	Gender	Age	Treatment	BW (kg)	BMI (kg/m^2^)	IS	Study Duration	Protocol	ΔBW (kg)	ΔBMI (kg/m^2^)	ΔIS (%)	IS Measure
Kelly et al. [51]	9 boys 11 girls	11.0	Control exercise	73.5 75.4	30.5 32.1	6.0 6.2	8 wk	4 times/wk, 30–50 min/session 50–80% of VO_2_ peak	0.8 1.1	−0.1 0.0	−2.8 5.2	2 h Glucose (OGTT) (mmol/L)

Meyer et al. [42]	47 boys 49 girls	12–16	Control exercise	NA	31.0 29.8	4.4 3.9	6 mo	3 days/wk, 60–90 min/day swimming, games, and so forth	NA	0.3 −2.6*	11.0 −20.8*	HOMA-IR

Kim et al. [52]	26 boys (Korean)	17.0	Control exercise	90.4 89.7	29.4 29.6	2.9 2.5	6 wk	5 days/wk, 40 min/day Jump rope (60–90 jumps/min)	0.0 −2.2*	−0.3 −1.0*	−18.2 −33.6*	HOMA-IR

Savoye et al. [19]	68 boys 106 girls	8–16	Control exercise	91.2 87.0	36.2 35.8	5.2 5.1	1 yr	Exercise & behavior modification. >100 min/wk for the first 6 mo >200 min/mo for the last 6 mo 65–80% of maximal HR	6 mo/1 yr 5.0/7.7 −2.6^†^/0.3^†^	6 mo/1 yr 1.1/1.6 −2.1^†^/−1.7^†^	6 mo/1 yr 6.1/16.8 −27.7^†^/−27.8^†^	HOMA-IR

Lee et al. [20]	45 boys	12–18	Control exercise	100.0 106.5	33.9 36.6	2.7 2.2	3 mo	3 days/wk, 60 min/session 50~75% of VO_2_ peak	2.6* −0.04	0.3 −0.3	−3.7 18.2^∗†^	Euglycemic clamp (mL/kg/min per *µ*U/min)

Davis et al. [21]	94 boys 128 girls	7–11	Control Low-dose exercise High-dose exercise	NA	26.3 25.9 25.6	2.5 2.2 2.4	10–15 wk	5 days/wk, HR > 150 bpm Low-dose: 20 min/day High-dose: 40 min/day	NA	NA	Adjusted mean diff. 0.11 (high versus low) 0.56 (low versus con.) 0.67 (high versus con.)	Matsuda index of insulin sensitivity

BMI: body mass index; BW: body weight; HOMA-IR: homeostasis model assessment of insulin resistance; IS: insulin sensitivity; OGTT: oral glucose tolerance test; Δ: Change score.

*Significantly different from baseline within each group (*P* < 0.05).

^†^Significantly different from controls or other groups (*P* < 0.05).

**Table 3:** Resistance or combined exercise and insulin resistance (nonrandomized controlled trials).

References	Gender	Age	Treatment	BW (kg)	BMI (kg/m^2^)	IS	Study Duration	Protocol	ΔBW (kg)	ΔBMI (kg/m^2^)	ΔIS (%)	IS Measure
Treuth et al. [46]	22 girls	7–10	Control (lean) exercise	29.1 46.6	NA	NA 315.9	5 mo	3 days/wk, 50% of 1RM 2 sets, 12–15 reps, 7 exercises	2.9* 4.0*	NA	NA −8.2	Insulin AUC (OGTT) (pmol/L)

Bell et al. [47]	8 boys 6 girls	12.7	Exercise	80.6	31.6	8.2	8 wk	3 days/wk, 60 min/session Strength exercise (2 sets, 10 exercises, 55–65% of 1RM) + Aerobic exercise (cycling)	0.6	−0.4	22.2*	Euglycemic clamp M_(lbm)_ (mg/kg/min)

Van der Heijden et al. [53]	6 boys 6 girls	15.5	Exercise	97.0	35.3	3.0	12 wk	2 times/wk, 60 min/session 2-3 sets, 8–20 reps	2.6*	0.8*	24.0*	Hepatic insulin sensitivity index

BMI: body mass index; BW: body weight; HOMA-IR: homeostasis model assessment of insulin resistance; IS: insulin sensitivity; OGTT: oral glucose tolerance test; RM: repetition maximum; Δ: Change score.

*Significantly different from baseline within each group (*P* < 0.05).

^†^Significantly different from controls or other groups (*P* < 0.05).

**Table 4:** Resistance or combined exercise and insulin resistance (randomized controlled trials).

References	Gender	Age	Treatment	BW (kg)	BMI (kg/m^2^)	IS	Study Duration	Protocol	ΔBW (kg)	ΔBMI (kg/m^2^)	ΔIS (%)	IS Measure
Shaibi et al. [22]	22 boys	15.6 15.1	Control exercise	98.3^†^ 90.0	34.6 32.5	1.7 2.3	16 wk	2 days/wk, <1 h/session 2 sets, ~15 reps, 10 exercises ~97% of 1RM	2.1 1.9	0.4 0.4	5.9 39.1^∗†^	FSIVGTT (×10^−4^/min/*µ*U/mL)

Chang et al. [54]	36 boys 13 girls (Chinese)	12–14	Control exercise	71.3 75.8	27.1 27.5	7.5 6.8	1 yr	2–5 times/wk, 60–90 min/session Aerobic + strength exercise 0–9-months: supervised exercise 10–12-months: no intervention	9 mo/1 yr *≈*4.5/7.5 *≈*1.2/6.2	9 mo/1 yr *≈*0.5/0.7 *≈*–0.6/0.6	9 mo/1 yr NA/44.0* −48.5*/44.1	HOMA-IR

Benson et al. [55]	46 boys 32 girls	10–15	Control exercise	51.7 59.2	22.1 24.0	1.1 1.3	8 wk	2 days/wk, 2 sets, 8 reps 11 exercises, 80% of 1RM	2.0 1.5	0.4 0.0	0.2 (log) 0.1 (log)	HOMA-IR

Davis et al. [56]	28 boys 26 girls	14–18	Control nutrition EDU (NU) NU + exercise	93.0 87.9 95.3	33.7 32.3 34.9	1.8 1.6 1.5	16 wk	2 days/wk, <1 h/session 2 sets, ~15 reps, 10 exercise ~97% of 1RM + 1 time/wk of nutrition EDU, 90 min/session	0.6 0.1 −0.3	0.2 −0.1 0.0	5.6 12.5 0.0	FSIVGTT (×10^−4^/min/*µ*U/mL)

Davis et al. [57]	44 youth	14–18	Control exercise Motivation + exercise	94.4 80.2 88.5	36.4 32.4 34.6	4.9 2.0 1.6	16 wk	2 days/week, 60–90 min/session 30–45 min of aerobic exercise (70–85% of maximal HR) + 30–45 min of strength exercise 8 motivation interview sessions	NA	NA	−4.0 −21.0 NA	HOMA-IR

Lee et al. [20]	45 boys	12–18	Control exercise	100.0 97.7	33.9 34.5	2.7 2.9	3 mo	3 days/wk, 60 min/session 1-2 sets, 8–12 reps, 10 exercises >60% of baseline 1RM	2.6* −0.6	0.3 −0.6*	−3.7 27.6^∗†^	Euglycemic clamp (mL/kg/min per *µ*U/min)

BMI: body mass index; BW: body weight; EDU: education; FSIVGTT: frequently sampled intravenous glucose tolerance test; HOMA-IR: homeostasis model assessment of insulin resistance; IS: insulin sensitivity; OGTT: oral glucose tolerance test; RM: repetition maximum; Δ: change score.

*Significantly different from baseline within each group (*P* < 0.05).

^†^Significantly different from controls or other groups (*P* < 0.05).
